# Characterization of Microbiota Associated with Digesta and Mucosa in Different Regions of Gastrointestinal Tract of Nursery Pigs

**DOI:** 10.3390/ijms20071630

**Published:** 2019-04-02

**Authors:** Bishnu Adhikari, Sung Woo Kim, Young Min Kwon

**Affiliations:** 1 Department of Poultry Science, University of Arkansas, Fayetteville, AR 72701 USA; bxa015@uark.edu; 2 Department of Animal Science, North Carolina State University, Raleigh, NC 27695, USA; sungwoo_kim@ncsu.edu; 3 Cell and Molecular Biology Program, University of Arkansas, Fayetteville, AR 72701, USA

**Keywords:** pig, gut microbiota, post-weaning stress, dysbiosis, diarrhea

## Abstract

Weaning is a crucial period when piglets have to cope with sudden dietary, social, and environmental stressors that often lead to serious intestinal dysbiosis and mortality. In this study, five mucosal and five digesta samples from each proximate jejunum, distal jejunum, and mid-colon were collected from 7- and 27-day post-weaned pigs and subjected to microbiota analysis using 16S rRNA gene profiling. Taxonomic analysis at phylum level revealed that Proteobacteria was significantly higher at 7 days (13.54%), while Bacteriodetes was higher at 27 days (30.72%) post weaning. Genera such as *Campylobacter*, *Veillonella*, *Helicobacter*, and *Blautia* that were previously reported in intestinal dysbiosis were significantly enriched in seven-day post-weaned pigs. However, microbial communities shifted as post weaning age increased with a significant increase in alpha diversity, and genera such as *Moryella*, *Dialister*, *Clostridium*, *Streptococcus*, *Prevotella*, and *Bacteroides* become significantly abundant in 27-day post-weaned pigs. Interestingly, the genus *Campylobacter* was significantly abundant on seven-day post-weaning in two piglets with diarrhea, implicating its role in post-weaning diarrhea. The results of this study suggest that gut microbiota in pigs with dysbiosis on 7-day post weaning undergoes significant changes toward a more normal state as the post-weaning age reaches 27 days.

## 1. Introduction

Along with the rapid advancement in the field of sequencing and bioinformatics, the microbiome studies of humans and other vertebrates have increased rapidly during the last decade. It is now an established fact that the gastrointestinal tract (GIT) of animals harbor complex and diverse groups of microbiota that affect the health and disease of the host. It is also widely accepted that the microbiota not only vary with different regions of GIT, but also with different locations (lumen vs. mucosa) within the same region [1,2,3]. Various factors including diets, antibiotics, infant feeding, illness, stress, aging, lifestyles, and host genetics have been found to affect gut microbiota [4,5,6,7,8]. Although very few as compared to humans, several studies have been conducted to characterize gut microbiota of pigs and reported differences in microbial composition among different regions and locations (mucosa vs. digesta) of GIT, which were further affected by pigs’ ages [1,9,10,11,12].

Among different periods of a pig’s life, weaning is a very critical period, which is commonly performed at 3–4 weeks of age, when piglets are separated from their mother and adapt to a new environment and diet [13]. This weaning age of pigs in the commercial swine industry is too early as compared to that of wild pigs, which normally occurs around 12 to 17 weeks after birth [14]. Since piglets have to cope with sudden dietary, social, and environmental changes during their early ages, they undergo severe stress that can lead to serious intestinal disorders, dysbiosis, and mortality [13,15,16]. Deaths due to gastrointestinal disorders that occur immediately post weaning were estimated to be around 17% of the total born in EU [15]. Because of this serious issue, antibiotics have been used commonly as both prophylactics and therapeutics in the swine industry, causing larger economic losses [17]. Moreover, because of the risk associated with antimicrobial resistance, use of antibiotics as growth performance parameters have already been banned in EU (EC Regulation No. 1831/2003) and restricted in several countries including the U.S. (FDA’s Guidance #213), demanding urgent search for antibiotic alternatives. Several alternatives to antibiotics such as probiotics, prebiotics, and essential oils have been evaluated in pigs and the studies suggest that modulation of gut microbiota might be one of their important mechanisms of action to exert beneficial effects on the host [18,19,20,21]. Thus, it is very important to understand the changes in gut microbiota at different ages of post-weaned pigs (nursery pigs) in order to develop strategies for modulation of gut microbiota to restore homeostasis or treat diseases associated with gut dysbiosis. Although previous studies have characterized pig gut microbiota, there is limited information regarding age-dependent distribution of gut microbiota in nursery pigs. In addition, microbiota residing on jejunum of pigs have been less characterized as compared to ileum. A recent study reported changes in microbiota composition and diversity during weaning transition, however this study was focused on fecal microbiota and all pigs were healthy [22]. In this study, we investigated the gut microbiota associated with digesta and mucosa at proximate and distal parts of jejunum and mid-colon at two different post-weaning ages (7 vs. 27 days) to provide a more comprehensive overview of gut microbiotas residing in GIT of nursery pigs (see Table 1). In addition, although few, some of the nursery pigs on day 7 post-weaning suffered from diarrhea, which enabled us to identify certain taxa strongly associated with diarrhea. 

## 2. Results 

### 2.1. Animals

Pigs showed normal growth pattern during the 27-day post-weaning feeding period. Average daily gain and average daily feed intake of 10 pigs during the first seven days were 63 ± 9 g and 88 ± 11 g per day, respectively. Average daily gain and average daily feed intake of five pigs during the entire 27 days were 330 ± 41 g and 478 ± 48 g per day, respectively. There was no mortality and no morbidity during the 27-day feeding period. 

### 2.2. Summary of Microbiota Data 

Summarization of Operational Taxonomic Unit (OTU) table resulted in 611,440 reads altogether, and the average reads per sample was 11,322 which ranged from 1949 to 43,273 reads per sample. More detailed information on the sequence reads is summarized in Table 1. The total number of features (OTUs) remained after the final data filtering step was 601.

### 2.3. Taxonomy Assignment at Phylum Level 

**Figure 1:**
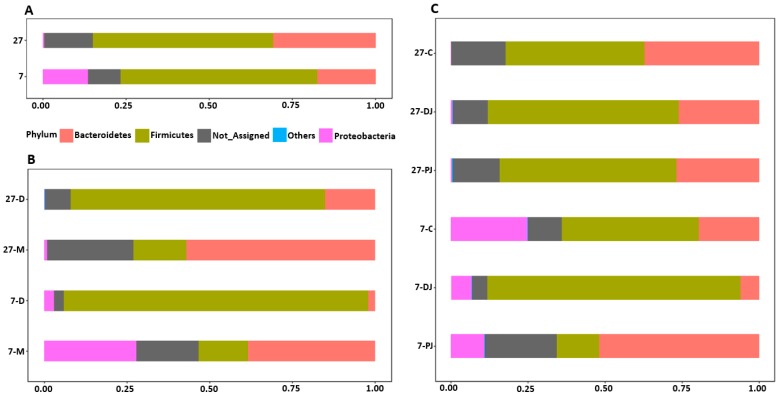
Relative abundance of different phyla at post weaning ages (**A**), different locations (**B**), and different regions of gastrointestinal tract (GIT) (**C**). The 7 and 27 represent 7 and 27 days, respectively, after weaning; M and D represent mucosa and digesta samples, respectively; PJ, DJ, and C represent proximate jejunum, distal jejunum, and mid-colon, respectively. Not_Assigned represents the sequence reads that were not assigned at the phylum level whereas Others represents the bacterial phyla whose counts were lower than 500.

On the taxonomic composition at phylum level, the relative abundance of Firmicutes (day 7, 59.14%; day 27, 54.23%) was found to be highest, followed by Bacteroidetes (day 7, 17.53%; day 27, 30.72%), and Proteobacteria (day 7, 13.54%; day 27, 0.34%) for both age groups (Figure 1A). Actinobacteria and Cyanobacteria were found less than 0.1%. As age increased, the relative abundance of Bacteroidetes and Cyanobacteria significantly increased, while the relative abundance of Proteobacteria and Actinobacteria significantly decreased (MetagenomeSeq, *p* < 0.05). In both ages, Firmicutes was dominant in digesta samples and was significantly different from mucosal samples, while Bacteroidetes was found predominant in mucosal samples and were significantly different from digesta samples (MetagenomeSeq, *p* < 0.05). Regarding Proteobacteria, it was found majorly on mucosal samples at seven days post weaning and was significantly different from digesta samples on both ages (MetagenomeSEq, *p* < 0.05) as shown in Figure 1B. Although there were no significant differences among different phyla present in three different regions on seven days post weaning, Bacteroidetes were found predominantly in proximate jejunum, whereas Firmicutes were found predominantly in both distal jejunum and mid-colon (MetagenomeSeq, *p* > 0.05). In addition, Proteobacteria was found to be highest in mid-colon followed by proximate jejunum and distal jejunum at seven days post weaning (Figure 1C). In contrast, Firmicutes were predominant in all three regions at 27 days post weaning and Proteobacteria was almost abolished in mid-colon. However, there were no significant differences among different phyla at three different locations at 27 days post weaning as on 7 days post weaning.

### 2.4. Genus Level

**Figure 2:**
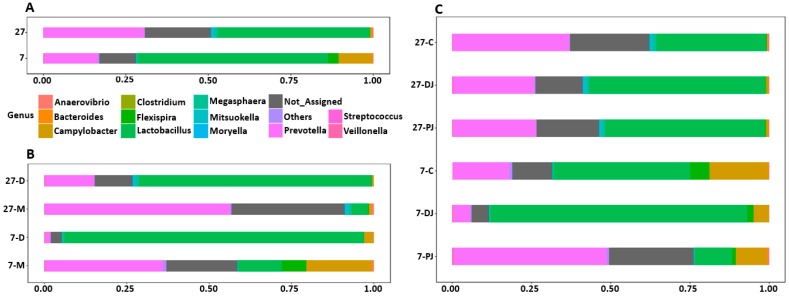
Relative abundance of different genera at post-weaning ages (**A**), different locations (**B**), and different regions of GIT (**C**). The 7 and 27 represent 7 and 27 days, respectively, after weaning; M and D represent mucosa and digesta samples, respectively; PJ, DJ, and C represent proximate jejunum, distal jejunum, and mid-colon, respectively. Not_Assigned represents the sequence reads that were not assigned at the genus level but assigned at the higher taxonomic level, whereas Others represents the bacterial phyla whose counts were lower than 500.

The relative abundance of different bacterial genera is shown in Figure 2. The bacterial genera with counts lower than 500 were grouped together into “Others”. Regardless of post weaning ages, *Lactobacillus* (7, 57.61% vs. 27, 46.05%) was found as a predominant genus followed by *Prevotella* (7, 16.12% vs. 27, 30.31%), as shown in Figure 2A. *Prevotella* was found as a predominant genus in mucosa of both post-weaning ages (7 days, 35.61%; 27 days, 56.29%). In contrast, *Lactobacillus* was found as a predominant genus in digesta of both ages (7 days, 90.92%; 27 days, 70.56%) as shown in Figure 2B. Regarding different regions across GIT, *Lactobacillus* and *Prevotella* were predominant genera in both post-weaning ages. At seven days post weaning, *Prevotella* was found predominantly in proximate jejunum (48.23%), while *Lactobacillus* was found predominantly in distal jejunum (80.92%) and mid-colon (43.02%), as shown in Figure 2C. In addition, *Campylobacter* was found to be highest in mid-colon (18.40%), followed by proximate jejunum (9.77%) and distal jejunum (4.69%). Similarly, *Flexispira* was found to be highest in mid-colon (6.01%), followed by distal jejunum (2.03%) and proximate jejunum (1.04%). In contrast, at 27 days post weaning, *Prevotella* was found predominantly in mid-colon (38.25%), while *Lactobacillus* was found predominantly in proximate (50.43%) and distal jejunum (55.54%). Both *Campylobacter* and *Flexispira* were present in much lower levels (<1%) at 27 days post weaning in contrast to 7 days post weaning.

### 2.5. Differentially Abundant Taxa at Two Different Ages, Locations, and Regions of GIT

At phylum level, regardless of different locations and regions, Proteobacteria and Actinobacteria were significantly abundant at 7 days post weaning, while Bacterioidetes and Cyanobacteria were significantly abundant at 27 days post weaning. Similarly, at genus level *Campylobacter*, genus CF231, *Veillonella*, *Helicobacter*, *Flexispira*, *Bifidobacterium*, *Succinivibrio*, *and Blautia* were significantly higher at 7 days post weaning as compared to 27 days post weaning (*p* < 0.05). In contrast, *Moryella, Mitsuokella*, *Dialister*, *Clostridium*, *Streptococcus*, *Prevotella*, *Megasphaera*, and *Bacteroides*, were significantly higher at 27 days post weaning as compared to 7 days post weaning (*p* < 0.05). In addition, although there were no significant differences, *Lactobacillus*, *Roseburia*, and *Coprococcus* were higher at 7 days post weaning, while *Anaerovibrio* and *Eubacterium* were higher at 27 days post weaning.

In addition, regardless of post-weaning ages, Fimicutes and Proteobacteria were found significantly higher in digesta, whereas Bacteroidetes were found significantly higher in mucosa. Likewise, at genus level, *Anaerovibrio*, *Bacteroides*, *Blautia*, *Campylobacter*, *Flexispira*, *Helicobacter*, *Prevotella*, *Roseburia*, and *Succinivibrio*, were significantly higher in mucosa as compared to digesta (*p* < 0.05). However, *Lactobacillus*, *Megasphaera*, *Clostridium*, and *Streptococcus* were found significantly higher in digesta as compared to mucosa (*p* < 0.05). 

**Figure 3:**
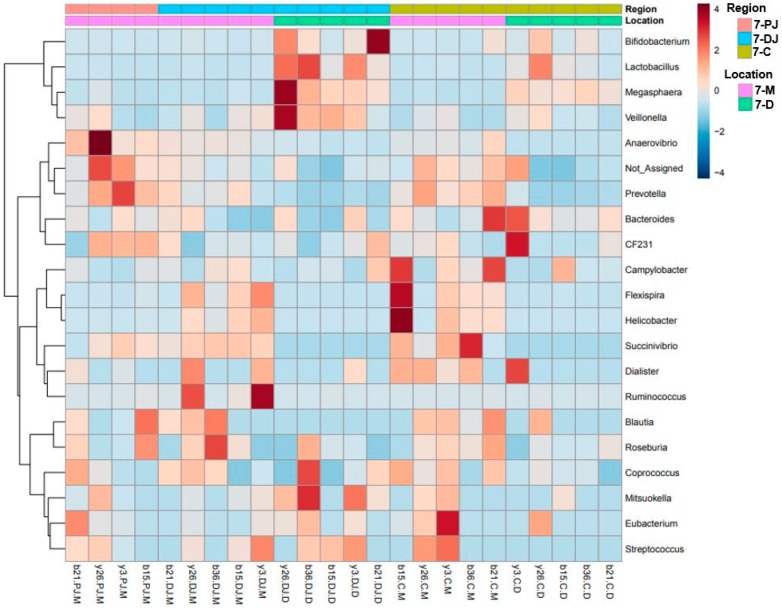
Hierarchical clustering heat map of bacterial genera from 7-day post-weaned pigs generated by MicrobiomeAnalyst using Euclidean distance measure and Ward clustering algorithm. The 7 represents 7 days post weaning; M and D represent mucosa and digesta samples, respectively; PJ, DJ, and C represent proximate jejunum, distal jejunum, and mid-colon, respectively. Not_Assigned represents the sequence reads that were not assigned at the genus level but assigned at the higher taxonomic level.

**Figure 4:**
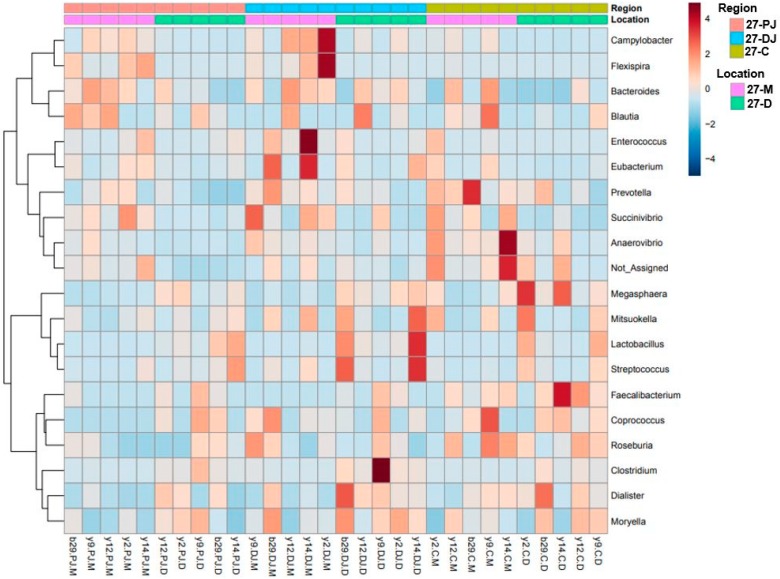
Hierarchical clustering heat map of bacterial genera from 27-day post-weaned pigs generated by MicrobiomeAnalyst using Euclidean distance measure and Ward clustering algorithm. The 27 represent 27 days post weaning; M and D represent mucosa and digesta samples, respectively; PJ, DJ, and C represent proximate jejunum, distal jejunum, and mid-colon, respectively. Not_Assigned represents the sequence reads that were not assigned at the genus level but assigned at the higher taxonomic level.

Furthermore, MetagenomeSeq identified *Prevotella*, genus CF231, *Anaerovibrio*, and *Roseburia* as differentially abundant genera across different regions regardless of the ages. All four genera were found highest in mid-colon as compared to proximate and distal jejunum, except that on day 7 these genera were found to be highest in proximate jejunum followed by mid-colon and distal jejunum. This may be due to the missing digesta samples from proximate jejunum on day 7, since they were found more abundant in mucosa as compared to the digesta samples. For better visualization of the differentially abundant bacterial genera across different regions and locations in 7- and 27-day post-weaned pigs, hierarchical clustering heat maps were created as shown in Figure 3 and Figure 4, respectively.

### 2.6. Core Microbiota on 7- and 27-Day Post-Weaned Pigs

**Figure 5:**
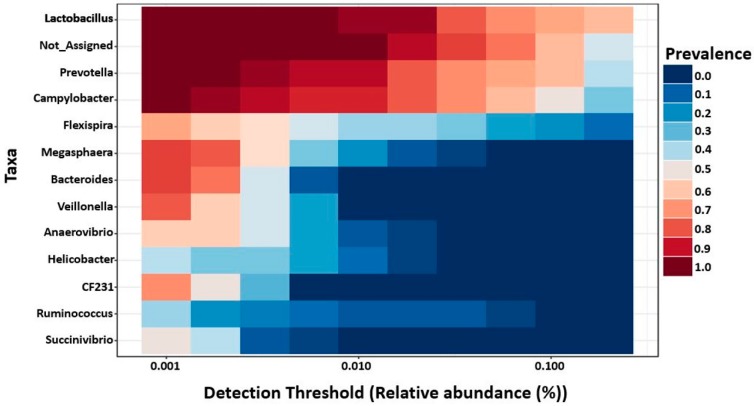
Core bacterial genera in 7-day post-weaned pigs as identified by MicrobiomeAnalyst using the parameters sample prevalence (20%) and relative abundance (0.2%). Not_Assigned represents the sequence reads that were not assigned at the genus level but assigned at the higher taxonomic level.

**Figure 6:**
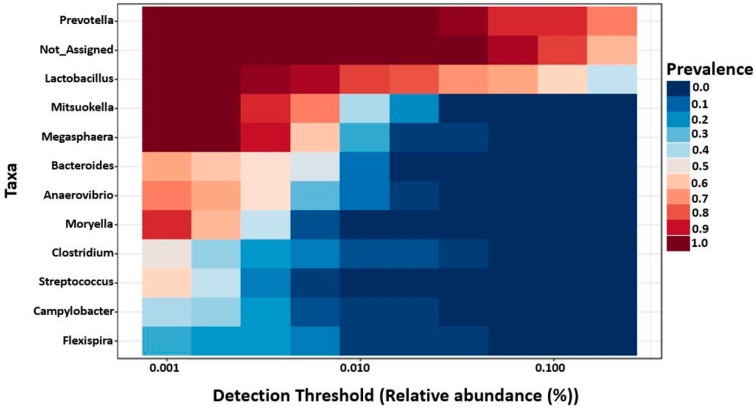
The core bacterial genera in 27-day post-weaned pigs as identified by MicrobiomeAnalyst using the parameters sample prevalence (20%) and relative abundance (0.2%). Not_Assigned represents the sequence reads that were not assigned at the genus level but assigned at the higher taxonomic level.

Core microbiome analysis was performed at genus level using MicrobiomeAnalyst based on sample prevalence and relative abundance cut off value at 20% and 0.2%, respectively. In 7-day post-weaned pigs, 12 core bacterial genera were identified as *Lactobacillus*, *Prevotella*, *Campylobacter*, *Flexispira*, *Megasphaera*, *Bacteroides*, *Veillonella*, *Anaerovibrio*, *Helicobacter*, genus CF231, *Ruminococcus*, and *Succinivibrio* in the descending order according to their prevalence in samples (Figure 5). In contrast, 11 core bacterial genera were identified in 27-day post-weaned pigs in the descending order of prevalence in samples as *Prevotella*, *Lactobacillus*, *Mitsuokella*, *Megasphaera*, *Bacteroides*, *Anaerovibrio*, *Moryella*, *Clostridium*, *Streptococcus*, *Campylobacter*, and *Flexispira* (Figure 6). 

### 2.7. Differentially Abundant Taxa in Piglets with Diarrhea

**Figure 7:**
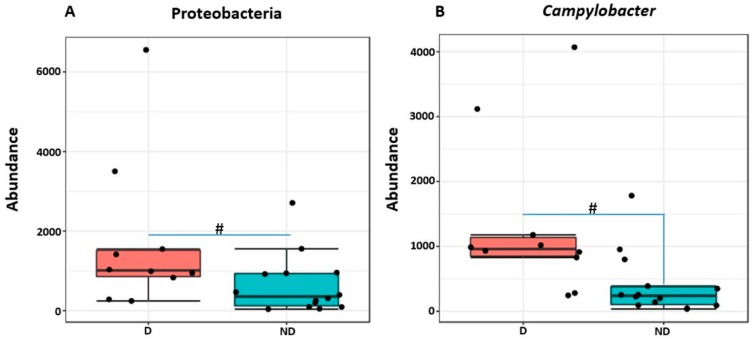
Abundance of Proteobacteria (**A**) and *Campylobacter* (**B**) in piglets with and without diarrhea. D and ND represent piglets with and without diarrhea, respectively. ^#^ represents significant difference between two groups at *p* < 0.05. Both Proteobacteria and *Campylobacter* were significantly increased in piglets with diarrhea as compared to healthy ones.

While comparing two groups of pig with or without diarrhea on day 7, we found significantly higher Proteobacteria and *Campylobacter* in the diarrheal group at phylum and genus levels, respectively, as shown in Figure 7 (MetagenomeSeq, *p* < 0.05). 

### 2.8. Alpha Diversity

**Figure 8:**
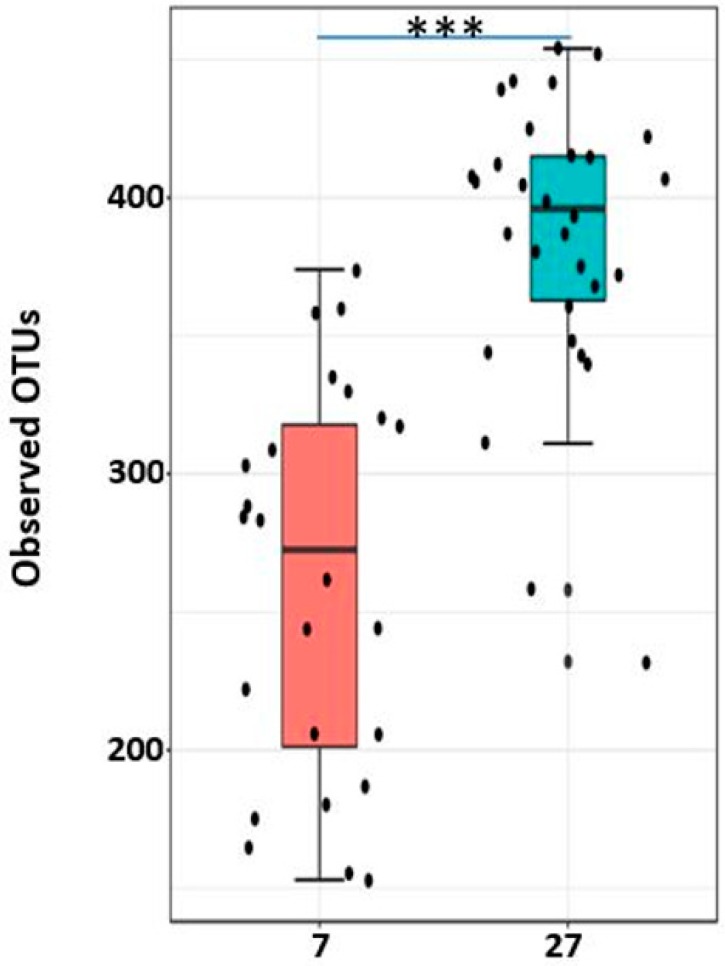
Alpha diversity between two post-weaning ages as measured by Observed_OTUS metric. *** represents significant difference between two groups at *p* < 0.0001. The 7 and 27 represent 7 and 27 days, respectively, after weaning. Increase in post-weaning age resulted an increase in alpha diversity.

**Figure 9:**
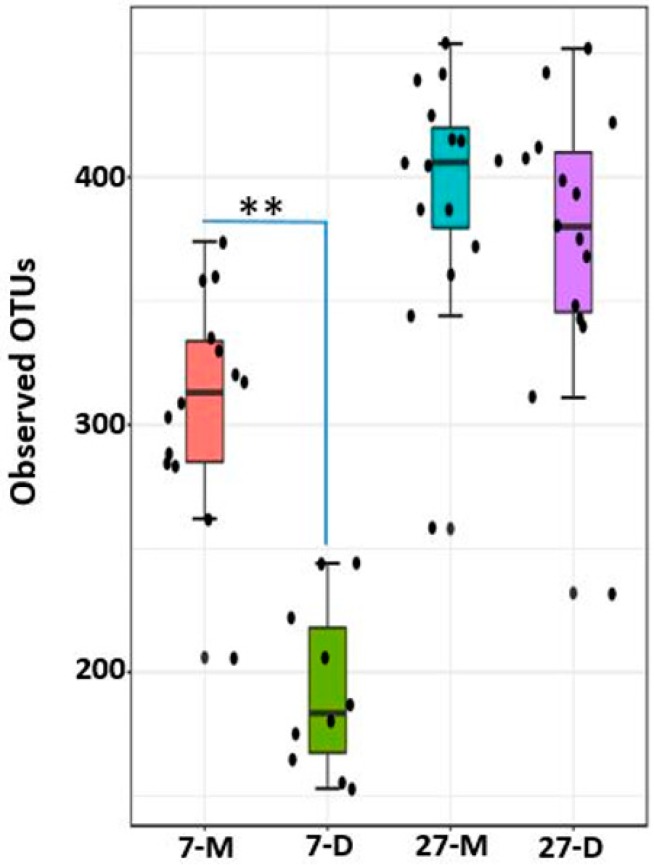
Alpha diversity between two different locations at two different post-weaning ages as measured by Observed_OTUS metric. ** represents significant difference between two groups at *p* < 0.001. The 7 and 27 represent 7 and 27 days, respectively, after weaning; M and D represent mucosa and digesta samples, respectively.

**Figure 10:**
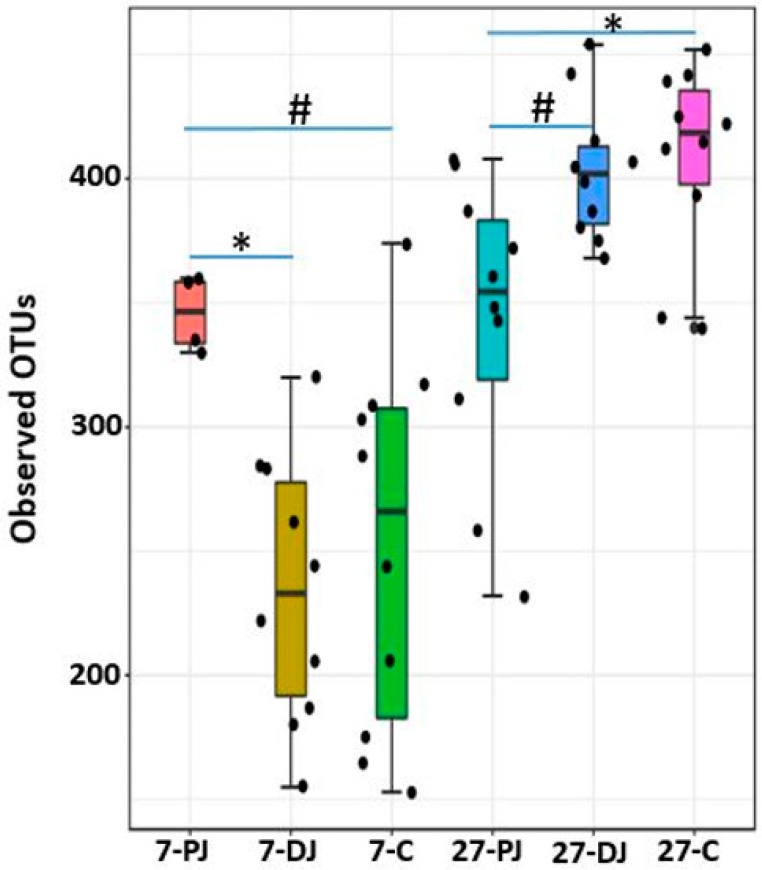
Alpha diversity between three different regions of GIT at two different post-weaning ages as measured by Observed_OTUS metric. ^#,^ * represent significant difference between two groups at *p* < 0.05 and *p* < 0.01, respectively. The 7 and 27 represent 7 and 27 days, respectively, after weaning; M and D represent mucosa and digesta samples, respectively; PJ, DJ, and C represent proximate jejunum, distal jejunum, and mid-colon, respectively.

The number of observed OTUs were in the range of 153–374 and 232–454 at day 7 and 27 post weaning, respectively. There was significant difference in alpha diversity (observed OTUs) between the two ages at *p* < 0.0001 as shown in Figure 8. The alpha diversity was higher in mucosa at both ages, however significant difference was observed only on day 7 post weaning (*p* < 0.001) as compared to the digesta, as shown in Figure 9. In addition, there was significant difference in alpha diversity among different regions of the GIT in both ages, as shown in Figure 10. On day 27, the alpha diversity in proximate jejunum, distal jejunum, and mid-colon was found in ascending order, where the alpha diversity was significantly higher in both mid-colon (*p* < 0.01) and distal jejunum (*p* < 0.05) as compared to the proximate jejunum. However, there was no significant difference between the two regions of jejunum. In contrast, the alpha diversity was the highest at proximate jejunum, followed by mid-colon on day 7 post weaning, while the significant differences were observed only in proximate jejunum as compared with distal jejunum (*p* < 0.01) and mid-colon (*p* < 0.05), as shown in Figure 10. 

### 2.9. Beta Diversity

**Figure 11:**
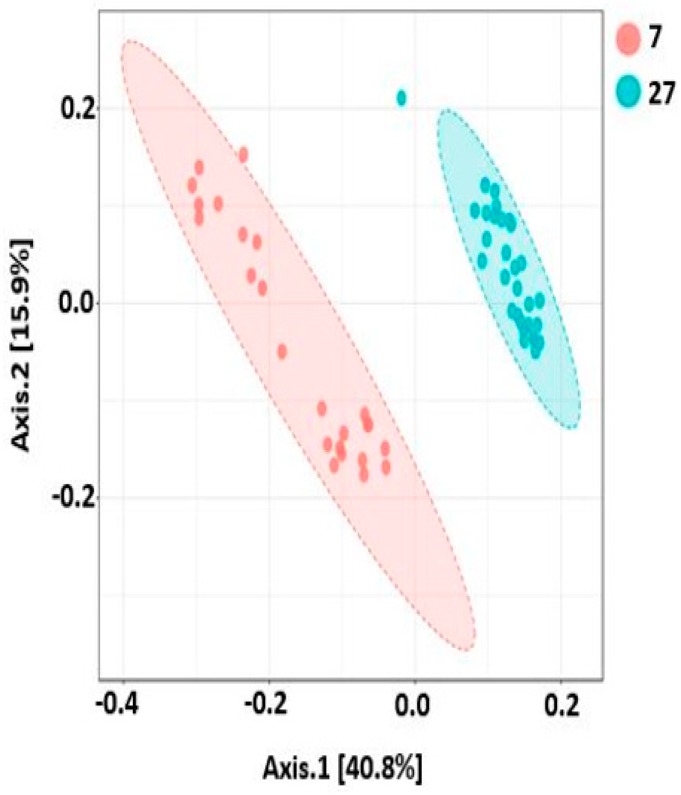
PCoA plot showing significant difference in bacterial community structure among piglets of two post weaning ages (ANOSIM; *R* = 0.76 and *p* < 0.001). Unweighted UniFrac distance metric was used to create PCoA plot. 7 and 27 represent 7 and 27 days respectively after weaning.

**Figure 12:**
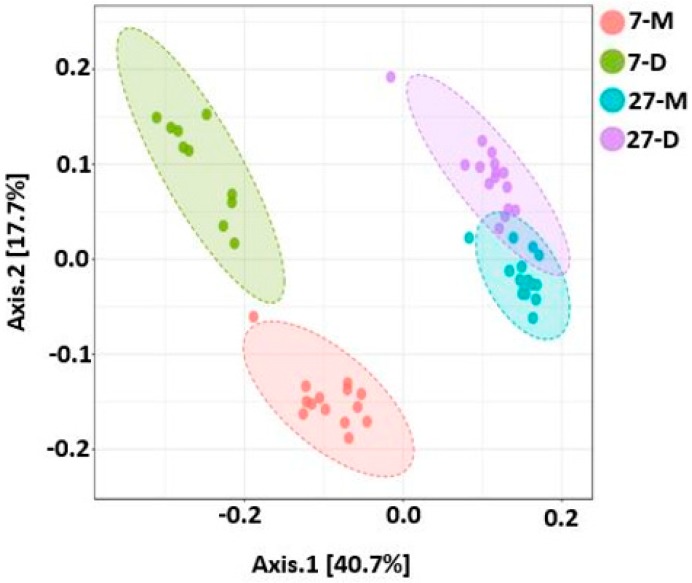
PCoA plot showing significant difference in bacterial community structure in different locations of GIT at two post-weaning ages of piglets (ANOSIM: *R* = 0.78, *p* < 0.001 for all four groups; *R* = 0.86, *p* < 0.001 for 7 days; *R* = 0.38, *p* < 0.001 for 27 days). Unweighted UniFrac distance metric was used to create PCoA plot. The 7 and 27 represent 7 and 27 days, respectively, after weaning; M and D represent mucosa and digesta samples, respectively.

**Figure 13:**
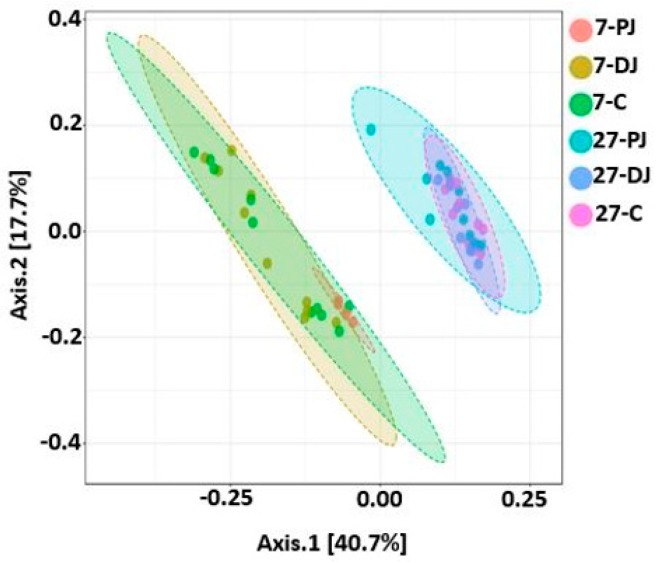
PCoA plot showing significant difference in bacterial community structure in three different regions of GIT at two post-weaning ages of piglets (ANOSIM: *R* = 0.45, *p* < 0.001). Unweighted UniFrac distance metric was used to create PCoA plot. The 7 and 27 represent 7 and 27 days, respectively, after weaning; M and D represent mucosa and digesta samples, respectively; PJ, DJ, and C represent proximate jejunum, distal jejunum, and mid-colon, respectively.

Like alpha diversity, there was significant difference in beta diversity between the two ages, which was clearly demonstrated by the ANOSIM results (*R* = 0.76 and *p* < 0.001), as shown in Figure 11. Similarly, the samples were significantly different between digesta and mucosa at both ages (all four groups, *R* = 0.78, *p* < 0.001; 7 days, *R* = 0.86, *p* < 0.001; 27 days, *R* = 0.38, *p* < 0.001), as shown in Figure 12. Similarly, the beta diversity was also significantly different when three different regions of both ages were compared (*R* = 0.45, *p* < 0.001) (Figure 13).

## 3. Discussion

In this study, we investigated the changes in microbiota composition and diversity of different regions and locations of GIT at two different post-weaning ages of pigs (Day 7 vs. 27). Regardless of post-weaning ages, Firmicutes were reported as a predominant phylum followed by Bacteroidetes, which is in agreement with previous studies [21,22]. In contrary, Pajarillo et al. reported Bacteroidetes as a predominant phylum in post-weaning pigs [23]. This variation may be due to difference in samples, breed, age, diets, and environments among different studies. Increases in post-weaning age significantly decreased the relative abundance of the phylum Proteobacteria. The phylum Proteobacteria contains many gram-negative pathogenic bacteria including *Escherichia*, *Salmonella*, *Campylobacter*, *Helicobacter*, and *Vibrio*, and its increase can be considered as a potential indicator of gut dysbiosis [24]. As mentioned earlier, weaning in pigs is a critical stage, where there are abrupt changes in environment and diets, and can lead to intestinal disorders. 

In this study, two pigs showed diarrhea at seven days post weaning, which may reflect the post-weaning stress of pigs. This may be one of the causes of gut dysbiosis associated with the increase in abundance of Proteobacteria. Increase in Proteobacteria at post-weaning ages was also reported earlier in pigs [10,21]. Proteobacteria was found significantly higher in mucosa as compared to lumen at both post-weaning ages. This is in agreement with previous finding where Proteobacteria was reported higher in colonic mucosa of both healthy and diseased adult (Swine Dysentery) [11] and nursery pigs [25]. In addition, Proteobacteria was also reported higher in mucosal samples (intestinal biopsy samples) than fecal samples in both healthy and diseased humans (inflammatory bowel disease; IBD) [26,27]. An increase in post-weaning age resulted in a concomitant increase in Bacteroidetes and decrease in Firmicutes. Age-dependent succession of bacterial communities in piglets were also reported previously, where the relative abundance of Bacteroidetes increased with the increase in age [28]. Like Proteobacteria, Bacteroidetes were also highly associated with mucosa, whereas Firmicutes were highly associated with lumen in both ages. In addition, the relative abundance of Bacteroidetes was the highest in mid-colon compared to both proximate and distal jejunum in 27-day post-weaned pigs as reported earlier [29]. However, in seven-day post-weaned pigs, Bacteriodetes were reported higher in proximate jejunum. This may be partially due to the missing digesta samples from proximate jejunum of seven-day post-weaned pigs, where Bacteroidetes were expected to be lower than in mucosa samples. Firmicutes were reported to be higher in both parts of jejunum at 27 days, while Prevotella was in mid-colon, especially at 7 days weaning. 

Interestingly, majority of genera such as *Campylobacter*, *Veillonella*, *Helicobacter*, *Succinivibrio*, and *Blautia* that were significantly enriched in seven-day post-weaned pigs were identified by Disbiome database as taxa that were previously reported in intestinal disorders (https://disbiome.ugent.be/home) [30]. As post-weaning age increased to 27 days, rapid changes in microbiota occurred, and bacterial genera such as *Moryella*, *Mitsuokella*, *Dialister*, *Clostridium*, *Streptococcus*, *Prevotella*, *Megasphaera*, *and Bacteroides* were significantly enriched and changed toward the normal gut microbiota of pigs. In addition, the core microbiota also differed between two different post-weaning ages, which further supports the changes in microbiota during weaning transitions. The core microbiota of 27-day post-weaned pigs resembled more to the core microbiota of typical swine gut [12], which suggests that the gut microbiota becomes stable as the post-weaning age increases.

In both ages, *Lactobacillus* and *Prevotella* were two major genera that were reported significantly higher in digesta and mucosa, respectively (*p* < 0.05). This is also reflected in the fact that Firmicutes (*Lactobacillus* belongs to this phylum) and Bacteriodetes (*Prevotella* belongs to this phylum) were higher in digesta and mucosa, respectively (*p* < 0.05). Likewise, *Anaerovibrio*, *Bacteroides*, *Blautia*, *Campylobacter*, *Flexispira*, *Helicobacter*, *Roseburia*, and *Succinivibrio* were significantly higher in mucosa, while *Megasphaera*, *Clostridium*, and *Streptococcus* were significantly higher in digesta (*p* < 0.05). This variation may be due to the difference in availability of oxygen, pH, and metabolites between lumen and mucosa of GIT which affect the composition of gut microbiota [31]. Furthermore, *Lactobacillus* and *Prevotella* were the two predominant genera of jejunum and colon especially at 27 days post weaning. A previous study also reported the same trend of *Lactobacillus* and *Prevotella* abundance in jejunum and colon of pigs [32]. On the contrary, although the trend was similar in the case of colon and distal jejunum at seven days post weaning, it did not match with the proximate jejunum which might be due to the missing mucosal samples from that particular region. 

Enterotoxigenic *Escherichia coli* proliferation is considered as one of the main causes of post-weaning diarrhea in pigs [33]. However, *Campylobacter* species, especially *C. coli* was also isolated from piglets and grower/finisher pigs with diarrhea [34,35]. In agreement with these studies, we also found a significant increase in *Campylobacter* in 7-day post-weaned pigs with diarrhea. Although this is a small study, it gives a new insight that not only *E. coli* but also *Campylobacter* can be an important causative agent of post-weaning diarrhea which is causing serious threats to swine industry. Furthermore, the alpha diversity increased as the post-weaning age increased and both alpha and beta diversity differed not only with different regions of the GIT, but also with different locations within the same region as reported earlier [1,12,21,25,32]. 

In sum, the overall results of the present study suggest that gut microbiotas undergo significant changes during the post-weaning period, becoming stable as the post-weaning age increases. Regardless of post-weaning age (7 vs. 27 days), the distribution and diversities of microbiota vary not only with different regions of the GIT, but also with different locations within the same region. In addition, significant abundance of *Campylobacter* in piglets with diarrhea suggest that the *Campylobacter* can also be one of the important etiological agents of post-weaning diarrhea. The findings of this study may provide aid for developing nutritional and management strategies to maintain balance of intestinal microbiota in nursery pigs. 

## 4. Materials and Methods

### 4.1. Animals

The animal experimental protocol used in this study was approved by North Carolina State University Animal Care and Use Committee. A total of 10 pigs (6.67 ± 0.46 kg, PIC genetics) that were weaned at 21 days of age were used in this study. Pigs were individually housed in a pen (1.2 × 2.0 m) with a single feeder and a single water nipple. Pigs had free access to feed and water. All pigs were fed to meet the nutrient requirements as suggested by National Research Council [36]. Corn (yellow dent), whey permeates (80% lactose), and poultry fat were major energy feeds. Soybean meal (dehulled), fish meal (menhaden), and blood plasma were major protein supplements. Body weight and the amount of feed consumed of each pig were recorded on day 7 and 27 after weaning. Five pigs were euthanized for the collection of digesta and mucosal samples on day 7 and 27 after weaning. 

### 4.2. Sample Collection and Processing

Immediately after euthanasia, digesta samples were aseptically collected from proximate jejunum (90 cm down from pyloric-duodenal junction), distal jejunum (60 cm up from ileo-cecal junction), and mid-colon (60 cm down from ileo-cecal junction). Mucosal samples were also collected from each respective region. Digesta and mucosal samples were snap frozen in liquid nitrogen and transferred and stored at −80 °C until DNA extraction. Thus, from 3 regions and 2 locations per region from each of 5 pigs, a total of 29 mucosal (one mucosal sample from proximate jejunum on day 7 was missing) and 25 digesta samples (proximate jejunal digesta samples from all five animals on day 7 were missing) were used in this study (Table 1). 

### 4.3. DNA Extraction and PCR

About 200 mg of each digesta and mucosal samples were used for genomic DNA extraction using QIAamp^®^ fast DNA stool mini kit following the manufacturer’s instructions. V1-V3 region of 16S rRNA gene from each genomic DNA sample was amplified by using unique barcoded universal primers as described previously [2]. The amplicons were purified from 0.7% agarose gel, and the concentration was measured using Qubit dsDNA broad range assay kit (Life Technologies, United States). Equal amounts of amplicons were pooled together, and gel-purified again from 6% TBE gel (Invitrogen, CA, United States). The final pooled sample was sequenced using MiSeq illumina 300 cycle paired-end options in Institute for Integrative Genome Biology at the University of California, Riverside (CA, U.S.). 

### 4.4. Data Analysis

All the MiSeq sequenced reads were checked for quality using FastQC [37] and low-quality reads were trimmed using Trimmomatic version 0.36 [38] with sliding window option 4:15. The quality filtered paired-end reads were joined together using join_paired_ends.py command of Quantitative Insights into Microbial Ecology, QIIME version 1.9.1 (available at: http://qiime.sourceforge.net) [39] with fastq-join option [40]. After joining, the barcodes positions were formatted using customized Perl script and the barcodes were removed using extract_barcodes.py command of QIIME. Split_libraries_fastq.py command of QIIME was used for demultiplexing of joined reads. The chimeric sequences were identified using USEARCH version 6.1.544 [41] and were excluded for downstream analysis. The OTU picking was performed using pick_open_reference_otus.py command of QIIME with uclust method [41]. OTUs that were found fewer than 2 times were discarded from the biom table for downstream analysis. Taxonomy was assigned based on green genes taxonomy and reference database version 13_8 [42] with RDP (Ribosomal Database Project) classifier [43]. For further statistical analysis and visualization, OTU table with taxa in plain format and metadata file were uploaded to the MicrobiomeAnalyst tool (available at: http://www.microbiomeanalyst.ca) [44]. Very low abundant features were filtered using options; minimum count 2 and low-count filter based on 20% prevalence in samples. Alpha diversity analysis was calculated based on Observed OTUs metric. Data were normalized using cumulative sum scaling before any statistical comparisons [45]. Significant differences in alpha diversity among different groups and pairwise comparisons were calculated based on Kruskal–Wallis/Wilcoxon tests using JMP Genomics 9 (SAS Institute Inc., Cary, NC). Significant difference level was set at *p* < 0.05. Beta diversity was calculated based on unweighted Unifrac distance metric [46] and statistical comparisons among groups were performed with analysis of similarities method (ANOSIM) [47]. The core microbiota analysis at two different post weaning ages was performed using MicrobiomeAnalyst with default parameters (20% sample prevalence and 0.2% relative abundance). In addition, hierarchical clustering heat map was created using the MicrobiomeAnalyst tool using Euclidean distance measure and Ward clustering algorithm. To determine differentially abundant phyla and genera among different groups, MetagenomeSeq [45] that uses zero-inflated Gaussian fit model was used with adjusted *p*-value cut off at 0.05. 

## Figures and Tables

**Table 1:** Summary of the sequence reads. NA refers to the missing samples.

Region	Location	No of Samples or Average Reads/Samples (Mean ± SE)	Days 7	Days 27
Proximate Jejunum	Mucosa	No. samples	4	5
		Reads/sample	10,064 ± 1781.96	5548.6 ± 715.70
	Digesta	No. samples	NA (missing)	5
		Reads/sample	NA (missing)	8588.8 ± 2275.10
Distal Jejunum	Mucosa	No. samples	5	5
		Reads/sample	4200.6 ± 458.17	10,382.6 ± 2622.45
	Digesta	No. samples	5	5
		Reads/sample	16,912.8 ± 5330.91	20,574.8 ± 8276.75
Mid Colon	Mucosa	No. samples	5	5
		Reads/sample	9156.6 ± 1178.52	15,440 ± 2789.66
	Digesta	No. samples	5	5
		Reads/sample	5975 ± 1928.96	17,698 ± 4778.06
